# The Cortical Actin Determines Different Susceptibility of Naïve and Memory CD4+ T Cells to HIV-1 Cell-to-Cell Transmission and Infection

**DOI:** 10.1371/journal.pone.0079221

**Published:** 2013-11-11

**Authors:** Marc Permanyer, Eduardo Pauls, Roger Badia, José A. Esté, Ester Ballana

**Affiliations:** AIDS Research Institute-IrsiCaixa, Hospital Germans Trias i Pujol, Universitat Autònoma de Barcelona, Badalona, Spain; New York University, United States of America

## Abstract

Memory CD4+ T cells are preferentially infected by HIV-1 compared to naïve cells. HIV-1 fusion and entry is a dynamic process in which the cytoskeleton plays an important role by allowing virion internalization and uncoating. Here, we evaluate the role of the cortical actin in cell-to-cell transfer of virus antigens and infection of target CD4+ T cells. Using different actin remodeling compounds we demonstrate that efficiency of HIV-internalization was proportional to the actin polymerization of the target cell. Naïve (CD45RA+) and memory (CD45RA−) CD4+ T cells could be phenotypically differentiated by the degree of cortical actin density and their capacity to capture virus. Thus, the higher cortical actin density of memory CD4+ T cells was associated to increased efficiency of HIV-antigen internalization and the establishment of a productive infection. Conversely, the lower cortical actin density in naïve CD4+ T cells restricted viral antigen transfer and consequently HIV-1 infection. In conclusion, the cortical actin density differentially affects the susceptibility to HIV-1 infection in naïve and memory CD4+ T cells by modulating the efficiency of HIV antigen internalization.

## Introduction

The HIV entry process is a validated target for antiretroviral therapy [1], [2]. However, different routes and mechanisms of infection of CD4+ T cells may contribute to the establishment of HIV reservoirs and increased HIV pathogenesis [3], [4]. Resting CD4^+^ T cells are the major reservoir of latent human immunodeficiency virus (HIV) infection and are a significant barrier to eradicating HIV because, upon stimulation, they are a source of viremia when antiretroviral therapy is interrupted [5]. Resting CD4+ T cells can be subdivided phenotypically into naïve and memory cell subsets as defined by the expression of multiple surface markers, including CD45RA, and depending on whether they have been previously exposed to a specific antigen. CD4+ memory T cells support higher levels of HIV replication than naïve CD4+ T cells, but the mechanism underlying the different susceptibility to HIV-1 infection remains unclear [6]–[8]. Memory resting CD4^+^ T cells differ from naïve resting CD4^+^ T cells in that they have a lower threshold for activation [9] and a subset of memory resting CD4^+^ T cells express higher levels of the HIV-1 coreceptor CCR5 than do naïve resting CD4^+^ T cells, while naïve cells express slightly higher levels of CXCR4 than memory cells [9]. However, the causes for the inherent resistance of naïve CD4+ T cells to HIV-1 infection cannot be explained by the different expression of viral coreceptors or the degree of activation of cells [8], [10]. Furthermore, although integrated proviral infection is found in both memory and naïve resting CD4+ T cells without the need of cell activation, integration in naïve cells was lower than that in memory cells, suggesting that restriction of infection occurs at the first steps of virus life cycle [10].

Several studies have shown that the viral dependence on the actin cytoskeleton during both early processes of infection, such as fusion and entry, but also at post entry steps, are required for the establishment of infection into CD4+ T cells [11]–[16] with a number of actin associated proteins regulating the role of cytoskeleton in viral entry [17]–[20]. Interestingly, a recent study found that the higher HIV-induced cortical actin dynamics in memory CD4+ T cells may promote efficient viral entry and viral DNA synthesis suggesting that phenotypic differences in the cortical actin between naïve and memory resting CD4+ T cells could account for the different cell susceptibility to HIV infection [8]. Additionally, cortical actin dynamics is also required during cell-to-cell HIV transmission by promoting the concentration of HIV antigens and its cellular receptors at the cell-cell contact zone [21]. Moreover, the uptake of HIV antigens into endocytic compartments after cell-to-cell transfer [22]–[25] could be prevented by pharmacological disruption of the cortical actin of effector cells [24], [26], [27], suggesting that active cytoskeleton dynamics is required for the internalization process. However, the role of the cytoskeleton during cell-to-cell HIV transmission into distinct T cells subsets has not been well characterized.

Here, we show that cell-to-cell transfer of HIV-1 antigens into primary resting CD4+ T cells is dependent on the polymerization of the cortical actin. Moreover, we show that phenotypic differences in the cortical actin in naïve and memory CD4+ T cells subsets determine the degree of viral antigen transfer inducing distinct susceptibilities to HIV-1 infection.

## Materials and Methods

### Ethics Statement

The work was approved by the scientific committee of Fundació IrsiCaixa. Human peripheral blood mononuclear cells were isolated from ‘buffy coats’ of healthy blood donors. Buffy coats were purchased anonymously from the Catalan Banc de Sang i Teixits (http://www.bancsang.net/en/index.html). The buffy coats received were totally anonymous and untraceable and the only information given was whether or not they have been tested for disease.

### Cells

Peripheral blood mononuclear cells (PBMC) from healthy donors were purified by Ficoll-Hypaque sedimentation. CD4+ T lymphocytes were immediately purified (>95%) from PBMCs by negative selection using the CD4+ T cell enrichment kit (Stem Cell Technologies, Vancouver, Canada) and grown in RPMI 1640 L-Glutamine medium (Gibco, Madrid, Spain) supplemented with 10% (R10) heat inactivated fetal calf serum (FCS, Invitrogen, Madrid, Spain), 100 U/ml penicillin, and 100 µg/ml streptomycin. When needed, CD4+ T cells were stimulated with phytohemagglutinin (PHA, Sigma, Madrid, Spain) at 4 µg/ml and 6 U/ml interleukin 2 (IL-2, Roche). MOLT-4 lymphoid cell line (AIDS Reagent Program, National Institutes of Health, Bethesda, MD) was cultured in R10. Chronically HIV-1-infected MOLT-4/CCR5 cells were generated after the infection of MOLT-4 cells, with the NL4-3 X4 HIV-1(MOLT_NL4-3_) [28], [29]. After the infection peak, the persistently infected culture was grown and characterized for Env expression and virus production. HEK293-T cells (AIDS Reagent Program, National Institutes of Health, Bethesda, MD) were cultured in Dulbecco’s modified Eagle’s medium (DMEM; Gibco, Madrid, Spain) supplemented with 10% heat inactivated FCS, 100 U/ml penicillin, and 100 µg/ml streptomycin.

### Cocultures of Infected and Uninfected Cells

Non-stimulated primary CD4+ T cells were cocultured with uninfected or HIV-1 persistently infected MOLT_NL4-3_ cells as previously described [22], [30], [31]. 2×10^5^ of both infected and target cells (1∶1 ratio) were cocultured in the absence or presence of 10 µg/ml anti-gp120 monoclonal antibody (mAb) IgGb12 (Polymun Scientific, Wien, Austria); 1 µg/ml reverse transcriptase (RT) inhibitor 3′-azido-3′-deoxythymidine (AZT) or 10 µg/ml CXCR4 coreceptor antagonist AMD3100 (both from Sigma-Aldrich). Cocultures were incubated overnight at 37°C in a 96-well culture plate in a final volume of 200 µl. Primary CD4+ T cells were pretreated with serial dilutions of Latrunculin-A (max. conc. = 1 µM), Phorbol 12-myristae 13-acetate (PMA, max. conc. = 15 nM) or Staurosporine (max. conc. = 1 µM) (all from Sigma-Aldrich) for 2 hours before coculturing for 4 hours with effector cells. Quantification of HIV-1 transfer was assessed by the percentage of CAp24-positive CD4+ T cells, using the coculture between primary T CD4+ lymphocytes and MOLT-4 uninfected cells as a control.

The use of cell lines as virus presenting cells has been extensively used by our group and others and is recognized as a useful cell culture model for cell-to-cell transmission [22], [24], [26], [31]–[36].

### Flow Cytometry

Cells were stained with CD4, CXCR4 (12G5) or CD45RA antibodies (BD Biosciences). Intracellular staining of HIV-1 p24 antigen (CAp24) was performed as previously described [22], [31]–[33]. Briefly, cells were fixed, permeabilized (Fix & Perm, Caltag, Burlingame, CA) and stained with the anti-HIV-CAp24 antigen mAb KC57 (Coulter, Barcelona, Spain). For F-actin staining, cells were fixed and permeabilized (Fix & Perm, Caltag, Burlingame, CA) and stained with 1 µg/ml of FITC-phalloidin (Sigma) for 30 min at RT in the dark. When needed, cells were first stained with surface CD45RA for 20 min before co-staining with intracellular CAp24, phalloidin or CCF2 for fusion assays (see below). Cells were analyzed in a LSRII flow cytometer (BD, Madrid, Spain) and identified by morphological parameters.

### Quantification of Cell-to-cell Transmission

2×10^5^ HEK293-T cells were cotransfected with 0,5 µg of HIV-1_NL4-3_ GFP (NIH AIDS Reagents Program). 48 h postransfection, HEK293-T cells were cocultured overnight with primary activated CD4+ T lymphocytes. HIV-antigen transfer into naïve (CD45RA+) and memory (CD45RA−) target cells was assessed by co-staining of surface CD45RA and intracellular staining of viral capsid p24 (CAp24). After overnight coculture cells were gently shaken to break cell-cell contacts and target CD4+ T cells were carefully harvested. 4 days after target cell purification productive infection was evaluated by GFP expression and assessed using flow cytometry.

### Virus-cell Fusion Assay

The quantification of the virus-cell membrane fusion was quantified as described before [25], [37]. Briefly, 2×10^5^ HEK293-T cells were cotransfected with 0,4 µg of both, the NL4-3 HIV provirus plasmid and a plasmid carrying the Vpr gene fused with beta-lactamase (Vpr-BlaM) (NIH AIDS Reagents Program). 48 h postransfection, HEK293-T cells were cocultured overnight with primary CD4+ T lymphocytes. Cells were loaded with the CCF2-AM loading kit (Invitrogen) following the protocol provided by the manufacturer. Cells were incubated 1 h at room temperature then washed and immediately fixed. The change in emission of the cleaved CCF2 generated by the BlaM-Vpr chimera was measured by flow cytometry.

## Results

### The Degree of Actin Polymerization Affects Cell-to-cell Transfer of HIV-1 Antigens

**Figure 1:**
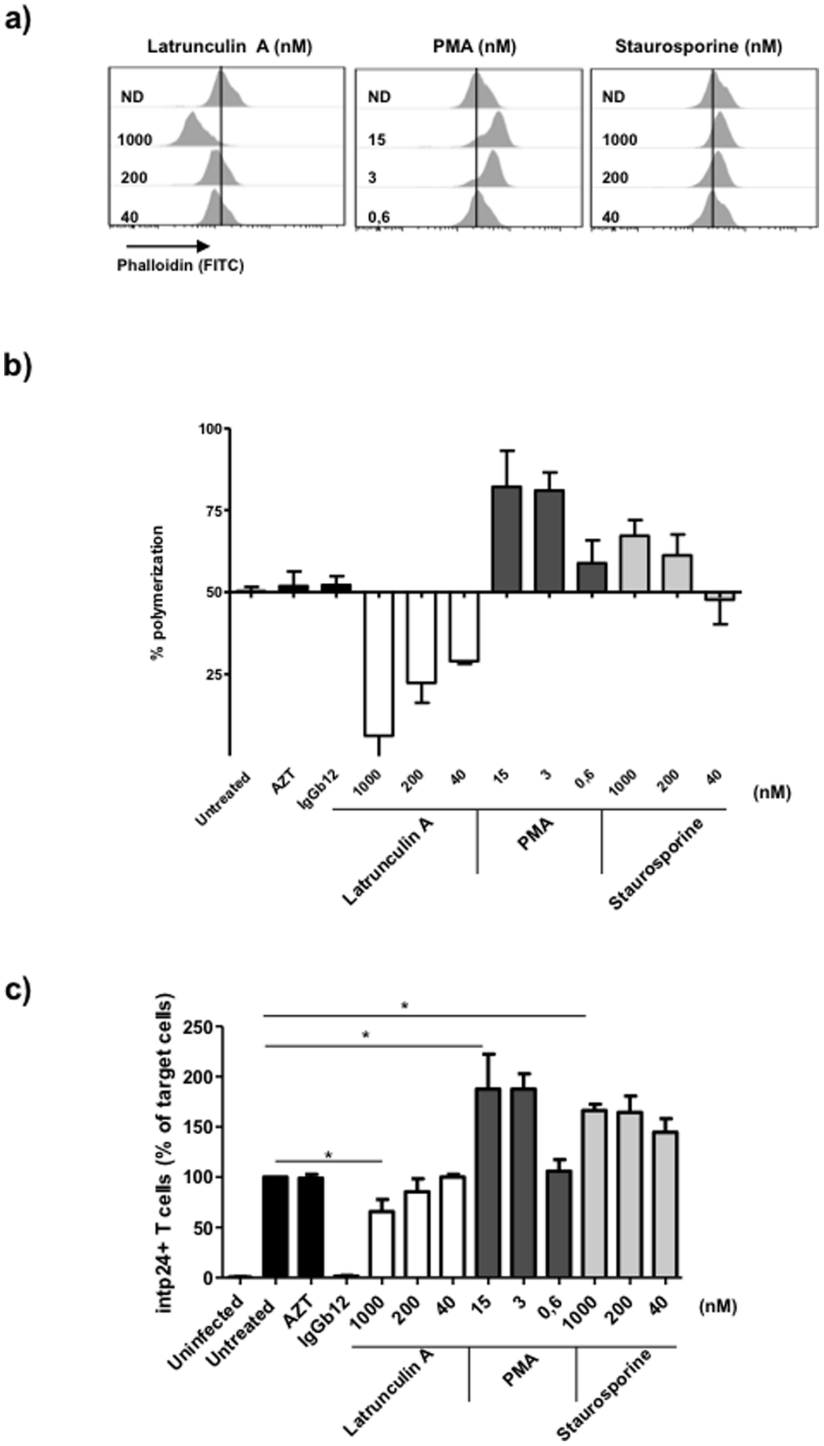
The cortical actin density modulates the transfer of HIV antigens during cell-to-cell contacts. Non-stimulated CD4+ T lymphocytes were pretreated with serial dilutions of latrunculin-A (max. conc. = 1 µM), PMA (max. conc. = 15 nM) or staurosporine (max. conc. = 1 µM) for 2 h. (a) Staining of F-actin with FITC-conjugated phalloidin was performed to assess the actin polymerization in treated cells. One representative experiment is shown. (b) The change in the percentage of actin polymerization normalized to the untreated condition. (c) Quantification of CAp24 HIV antigen transfer from infected to uninfected CD4+ T cells assessed by intracellular CAp24-antigen staining and analysed by flow cytometry. Control coculture condition were performed in the presence of IgGb12 (10 µg/ml) and AZT (1 µg/ml). The percentage of intracellular CAp24+ target cells was normalized to the untreated condition. Results are the mean ± SD of three independent experiments (*p<0.05).

Contacts formed between HIV-1 infected and uninfected primary CD4+ T cells induce the transfer of HIV antigens into endocytic compartments in the absence of fusion or infection [22]–[25], [31]. To evaluate the role of the cortical actin cytoskeleton of target cells during cell-to-cell HIV-1 antigen transfer, previously purified primary non-stimulated CD4+ T lymphocytes were pretreated for 2 hours with different actin remodelling compounds. After drug treatment, the degree of actin polymerization was assessed by intracellular F-actin staining (Fig. 1a and 1b). Transient treatment of resting CD4+ T cells with latrunculin-A triggered actin depolymerisation (45% reduction in the percentage of polymerization at 1 µM compared to the untreated condition). Conversely, treatment with PMA triggered actin polymerization (roughly 30% increase in the percentage of polymerization at 15 nM compared to the untreated condition), consistent with a previous report [38]. Furthermore, another approach to induce actin remodelling is modulating the cofilin activity by affecting the upstream signalling using different compounds such as staurosporine [16]. However, unlike a previous report [16], we observed a slight increase of actin polymerization in staurosporine-treated cells (17% increase in the percentage of polymerization compared to the untreated condition). Drug treatment did not cause a significant change in the expression of the cellular CD4 receptor or CXCR4 coreceptor (data not shown).

To evaluate the effect of the cortical actin remodelling in the transfer of viral antigens, drug pretreated primary CD4+ T cells were cocultured with HIV-1_NL4-3_ persistently infected MOLT_NL4-3_. After 4 hours of coculture, HIV antigen transfer to target cells was assessed by intracellular staining of CAp24 (Fig. 1c). Compared to the untreated condition, the transfer of viral antigens to uninfected cells was clearly blocked by the neutralizing anti-gp120 mAb IgGb12 (>95% inhibition), but was not inhibited by the RT inhibitor AZT indicating a CD4-dependent transmission of high amounts of HIV-1 particles in the absence of productive infection. Furthermore, compared to the untreated condition, the transfer of viral antigens into target CD4+ T cells decreased in latrunculin A pretreated cells (40% of inhibition of CAp24+ cells at 1 µM of latrunculin A) but increased in PMA or staurosporine pretreated cells (186% and 150% increase of CAp24+ cells, respectively at the highest concentrations tested). The lack of a more potent impact probably reflects the loss of effect over time due to the absence of the drug during the coculture. Taken together, these results demonstrate that the transmission of HIV antigens into target primary CD4+ T cells during cell-to-cell transfer is modulated by their degree of actin polymerization.

### Naïve and Memory CD4+ T cells Display Distinct Degree of Actin Polymerization

**Figure 2:**
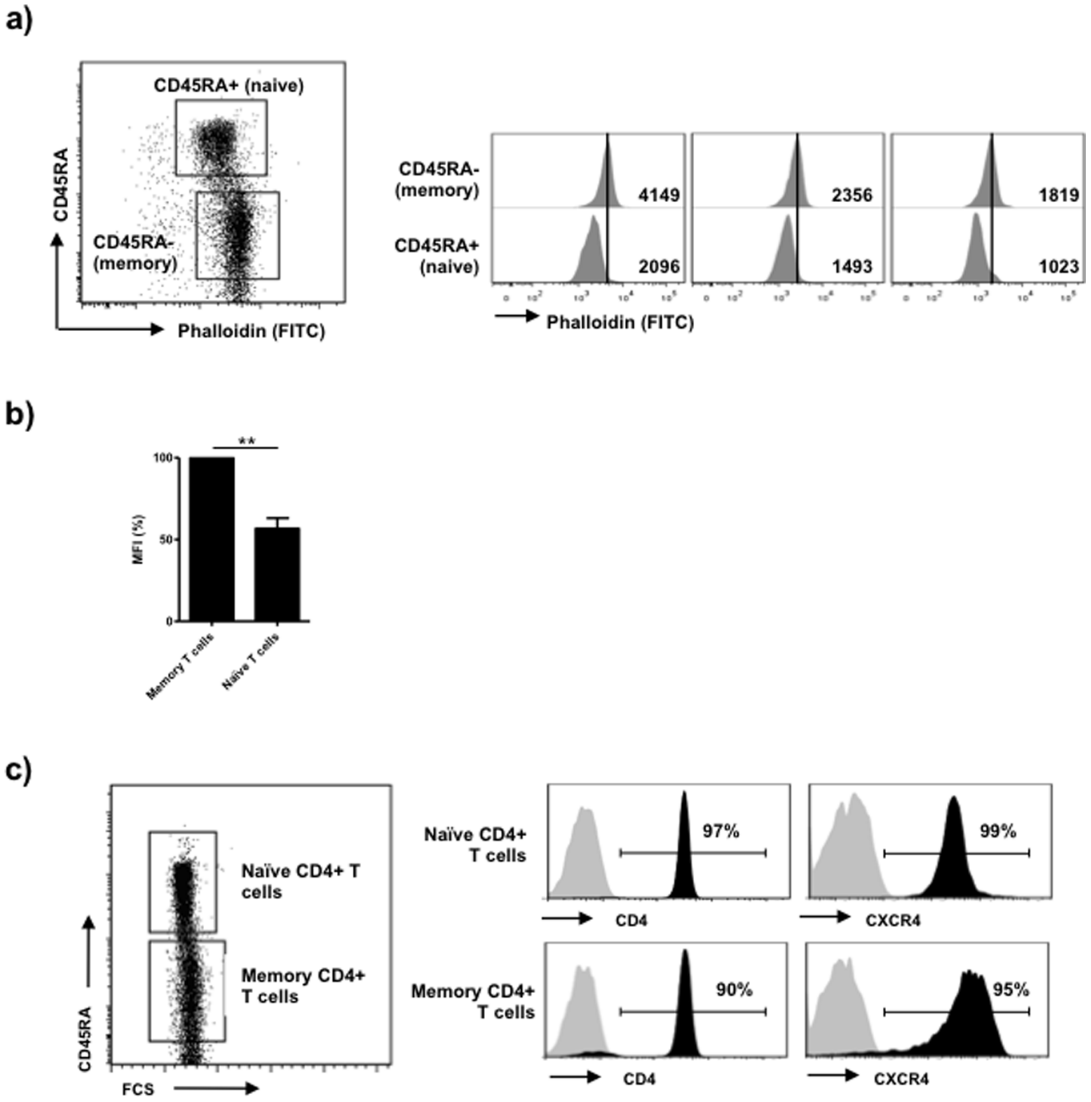
Distinct degree of cortical actin polymerization in naïve and memory CD4+ T cells. Resting CD4+ T cells were purified from peripheral blood by negative depletion. Surface expression of CD45RA differentiated between naïve (CD45RA+) and memory (CD45RA-) CD4+ T cells. (a) The F-actin in memory and naïve CD4+ T cells from 3 representative donors were evaluated by co-staining of CD45RA and FITC-labelled phalloidin and assessed by flow cytometry. (b) The MFI of F-actin of naïve and memory CD4+ T cells is plotted. Values are normalized to the memory T cell subset. Mean and SD of 3 different donors is shown (**p<0.005). (c) CD4 and CXCR4 receptor expression in naïve and memory CD4+ T cells from one representative donor is shown.

Several post-entry cellular mechanisms may explain the different susceptibility to HIV infection between naïve and memory CD4+ T cell subtypes [6]–[8], [39]. Because the cortical actin polymerization modulates the internalization of viral antigens during cell-to-cell contacts, we asked whether differences in cortical actin polymerization in distinct CD4+ T cell subtypes may determine different susceptibilities to infection by regulating the efficiency of viral antigen internalization. Naïve and memory CD4+ T cell subpopulations can be identified by the expression of surface CD45RA and CD45RO isoforms respectively. Thus, we performed co-staining of F-actin and surface CD45RA in primary non-stimulated CD4+ T cells to study the cortical actin polymerization of these two T cell subsets. In all donors evaluated, the intensity of the F-actin staining was higher in memory CD4+ T cells (Fig. 2a and 2b), indicating that memory CD4+ T cells display a more polymerized actin cytoskeleton than naïve CD4+ T cells. To discard that the different susceptibility to HIV-infection in both T cell subtypes could be determined by differences in the coreceptor expression, we evaluated the expression levels of CD4 and CXCR4 receptors in naïve and memory resting CD4+ T cells (Fig. 2c). As expected, we did not observe significant differences in both receptors, consistent with previous reports [6], [10].

### Higher Efficiency of HIV-1 Antigen Internalization into Memory CD4+ T cells during Cell-to-cell Transfer

**Figure 3:**
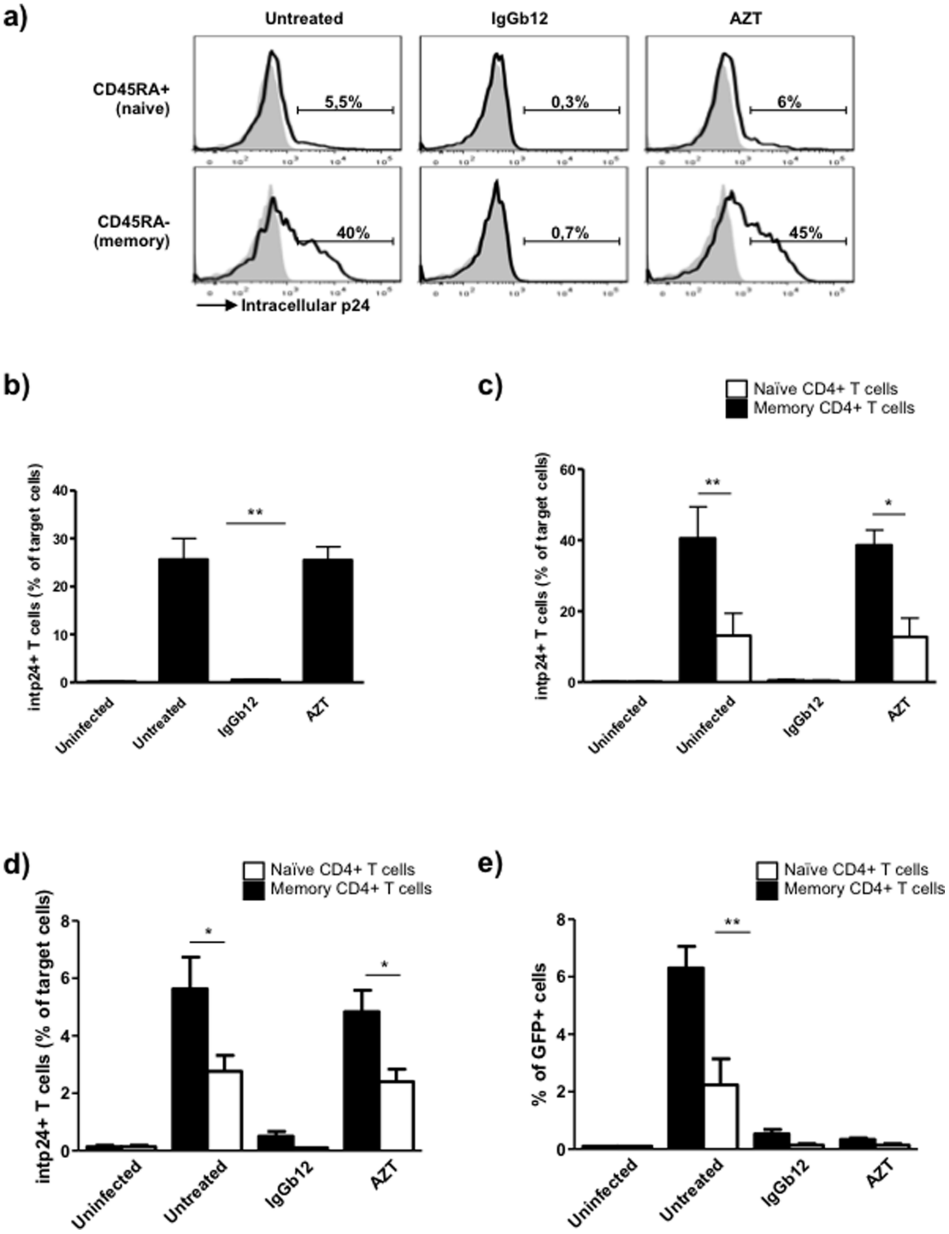
Different HIV-antigen internalization into naïve and memory CD4+ T cells. (a–c) Uninfected or infected MOLT_NL4-3_ cells were cocultured overnight with non-stimulated primary CD4+ T lymphocytes in the presence or the absence of the anti-HIV-1 gp120 mAb IgGb12 (10 µg/ml) or the RT inhibitor AZT (1 µg/ml). Quantification of CAp24 HIV antigen transfer from infected to naïve and memory CD4+ T cells was assessed by co-staining of CD45RA and intracellular CAp24-antigen staining and analysed by flow cytometry. (a) One representative experiment is shown. (b) Percentage of intracellular CAp24+ into total target CD4+ T cells. (c) Percentage of intracellular CAp24+ into naïve (CD45RA+) and memory (CD45RA−) CD4+ T cells. (d–e) HEK293-T cells cotransfected with HIV-1_NL4-3_ GFP plasmid were cocultured with primary activated CD4+ T cells in the presence or the absence of the anti-HIV-1 gp120 mAb IgGb12 (10 µg/ml) and the RT inhibitor AZT (1 µg/ml). After overnight coculture target cells were carefully harvested after gently shaken and cultured for 4 days in the same inhibitors. (d) Percentage of intracellular CAp24+ into naïve (CD45RA+) and memory (CD45RA−) CD4+ T cells after overnight cocultures. (e) Percentage of naïve (CD45RA+) and memory (CD45RA−) GFP+ cells 4 days after purification of target cells. Mean and SD of three independent experiments is shown (**p<0.005, *p<0.05).

Given that the internalization of HIV antigens by target cells is sensitive to the degree of cortical actin polymerization, we hypothesized that the different cortical actin density in naïve and memory CD4+ T cells may induce differences in the uptake of HIV antigens during cell-to-cell transfer. To evaluate the degree of HIV antigen internalization into naïve and memory CD4+ T cell subsets, cocultures of MOLT_NL4-3_ cells and primary resting CD4+ T cells were evaluated by flow cytometry. The transfer of viral antigens to total target CD4+ T cells (Fig. 3a and 3b) was clearly blocked by the neutralizing anti-gp120 mAb IgGb12 (>95% of inhibition compared with the untreated condition), but was not inhibited by the RT inhibitor AZT (23% of p24+ cells). As previously described [22], [23], [25], these results indicated a CD4-dependent transmission of high amounts of HIV-1 particles from infected to uninfected cells in the absence of fusion or infection. On the other hand, in all conditions the uptake of HIV particles by memory CD4+ T cells was significantly increased in untreated (p<0,005) or AZT-treated (p<0,05) target CD4+ T cells (10% and 35% of p24+ cells respectively), compared to naïve target CD4+ T cells (Fig. 3a and 3c), consistent with a previous report [36]. These results indicate that phenotypic differences between naïve and memory CD4+ T cells establish different affinities for HIV antigens during cell-to-cell viral antigen internalization, suggesting that the susceptibility to HIV infection may be determined during early internalization processes that may be related to cortical acting polymerization.

### Higher Efficiency of Cell-to-cell Transmission of HIV-1 into Memory CD4+ T cells

The susceptibility to HIV infection of naïve and memory CD4+ T cells has not been previously evaluated in the context of cell-to-cell transmission which is considered to propagate HIV infection more efficiently than cell-free virus spread [40], [41] through endocytic internalization of viral antigens in the absence of virus-cell fusion or infection [22]–[25], [31]. To assess the cell-associated transmission of HIV antigens into naïve and memory CD4+ T cells, activated primary CD4+ T cells were cocultured with HEK293T cells previously transfected with HIV-1_NL4-3_ GFP. This system allowed us to evaluate in parallel the non-productive transfer of viral antigens and the subsequent establishment of a productive infection. HIV-antigen transfer into naïve (CD45RA+) and memory (CD45RA-) target cells was assessed by intracellular staining of viral capsid p24 (CAp24) after overnight coculture (Fig. 3d) and productive infection was evaluated by GFP expression 4 days after target cell purification (Fig. 3e). As shown before, the transfer of viral antigens to uninfected cells was clearly blocked by the neutralizing anti-gp120 mAb IgGb12 (90% and 95% inhibition compared to the untreated condition in memory and naïve CD4+ cells respectively), but was not inhibited by the RT inhibitor AZT. After target cell purification, cells were left in culture in the presence of the same compounds. 4 days after culturing, infection of memory CD4+ T cells increased roughly 3-fold compared to naïve CD4+ T cells (6% and 2% of GFP+ cells in memory and naïve CD4+ T cells respectively) (Fig. 3e) consistent with previous results [6], [7], [42]. As expected, IgGb12 and AZT effectively block virus replication after cell-to-cell transmission [43]. Altogether, these results indicate that when cells are permissive to infection, virus replication is in concordance to the amount of virus transferred during the coculture phase suggesting that the susceptibility to HIV infection may be determined during early internalization processes even before viral entry.

### Viral Entry is not Restricted in any of both T cell Subsets

**Figure 4:**
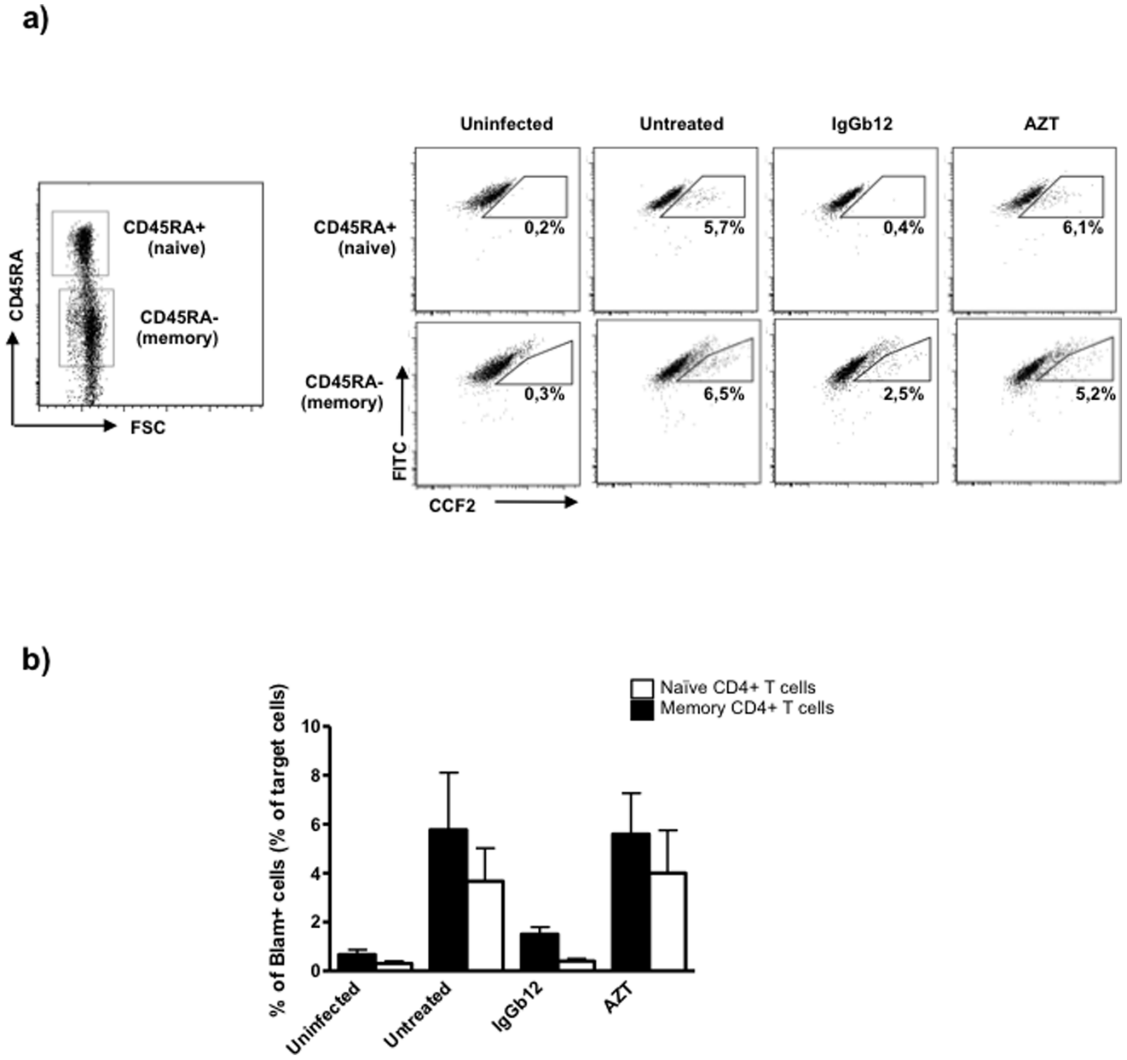
Similar levels of viral fusion into naïve and memory CD4+ T cells. HEK293-T cells cotransfected with pNL4-3 and BlaM-Vpr plasmids were cocultured with primary resting CD4+ T cells in the presence or the absence of the anti-HIV-1 gp120 mAb IgGb12 (10 µg/ml) and the RT inhibitor AZT (1 µg/ml). Viral fusion was assessed by flow cytometry by measuring the percentage of CCF2-cleaved naïve (CD45RA+) and memory (CD45RA−) target CD4 T cells. (a) Dot plots of CCF2-loaded cells (FITC-labelled) versus CCF2-cleaved cells (Pacific blue-labelled) of a representative experiment are shown. (b) Percentage of CCF2-cleaved target cells normalized to memory CD4+ T cells in untreated condition. Mean and SD of two independent experiments is shown.

Discrepant results have been reported regarding viral fusion of HIV-1 into naïve and memory T cell subsets [8], [10]. One previous report showed that naïve T cells were restricted at viral fusion [10], while the other study found only slightly diminished viral fusion in naïve T cells in one of two assays [8]. To find out whether HIV antigens are prevented from entering a subset of CD4+ target cells after cell-to-cell transfer, we evaluated the efficiency of viral entry in both T cell subtypes using the Vpr-β-lactamase-based entry assay [37]. HIV_NL4-3_ transfected *Vpr*-BlaM+ HEK293-T cells were cocultured with primary resting CD4+ T cells and fusion was measured in naïve (CD45RA+) and memory (CD45RA−) T cell subtypes by detection of the enzymatic cleavage of CCF2 dye using flow cytometry (Fig. 4a). We found that viral entry into naïve CD4+ T cells was reduced roughly 25% compared to memory CD4+ T cells but the difference was not statistically significant (Fig. 4b). Viral fusion into naïve CD4+ T cells was significantly inhibited by IgGb12 (95% reduction). Surprisingly, IgGb12 did not block viral fusion into memory CD4+ T cells as efficiently as into naïve CD4+ T cells (70% of inhibition compared to the untreated condition) (Fig. 4b). As expected, cleavage of CCF2 was not prevented by AZT. Taken together, these results indicate that after cell-to-cell transfer, viral entry is not restricted in any of the CD4+ T cells subtypes.

## Discussion

Actin polymerization but also other factors have been proposed to contribute to the different HIV susceptibility of naïve and memory T cells, especially expression of cell surface proteins, such as viral coreceptors, or the degree of activation of cells [8], [10], [44]. Here, we show that cortical actin density plays a prominent role in determining susceptibility to HIV-antigen capture and infection, mapping the restriction at early steps of viral life cycle after virus-cell fusion.

Our results are consistent with previous reports showing that the differential susceptibility in naïve and memory CD4+ T cells can already be detected during the initial stages of viral infection such as viral entry or DNA synthesis [8], [10], [39], [42]. Interestingly, differences in the cortical actin between naïve and memory CD4+ T cells affecting viral DNA synthesis have been recently reported in cell-free virus infections [10]. Unlike cell-free virus infection, our short-term coculture model between HIV-infected and non-stimulated primary CD4+ T cells maximise the cell-to-cell endocytic antigen transfer, which might determine differences in the infection outcome. There exist several evidences of the importance of viral entry route in relation to cytoskeleton remodelling and establishment of HIV infection. Viral entry via endocytosis may allow viruses to overcome the restriction of a static cortical actin or to evade antibody neutralization [45]. Moreover, it has been shown that cell-free and cell-to-cell HIV-1 infections were not equally sensitive to the actin inhibitor cytochalasin D [24] and, neither resting nor activated T cells are equally susceptible to infection by VSV-G-mediated endocytosis [46]. We have previously demonstrate that transferred HIV particles resurface to the outer cell membrane of resting CD4+ T cells, suggesting that endocytic uptake may serve as an itinerant virus reservoir capable of inducing trans-infection of cells after the release of HIV particles to the extracellular environment, but being unable to establish productive infection [3], [22], [25]. Thus, the infection system, the cell type or the activation state of the target cell may also condition the entry route [3], which simultaneously may impose different cytoskeleton requirements.

Reduced levels of virus-cell fusion in naïve CD4+ T cells were suggested to be responsible for the restriction in this subset of T cells [10], an observation that is in clear contrast with our results showing no significant differences in virus-cell fusion between memory and naïve T cells. Probably, the discrepancy between this report and our observations may arise from the different infection systems used. The higher efficiency of cell-to-cell HIV transmission compared to cell-free virus infection [4], [40], [41] may allow to overcome restrictions observed during cell-free virus infection and thus may minimise the qualitative differences between both T cell subtypes. Taken together, our results are in accordance to the findings reported in by Wang et al., [8]. Both conclude that cortical actin polymerization determines the susceptibility to infection during the steps prior to viral entry, through modulating the uptake of HIV-antigens during cell-to-cell transfer. It cannot be discarded that cytoskeleton remodelling may have an effect over other cellular processes involved in the viral infection cycle such as reverse transcription or budding as suggested by others [8], [27]. Similarly to cell-free virus infection [11]–[14], during cell-to-cell transfer there is an *Env*-induced actin-dependent HIV-receptor clustering at the cell-cell interface [21]. Thus, the different degree of actin polymerization in naïve and memory CD4+ T cells may induce distinct cellular receptor recruitment in both T cell subtypes, which in turn, may affect the efficiency of HIV-antigen internalization [47], in both cell-free and cell-to-cell virus infection.

Eradication of HIV-1 with antiretroviral therapy is not possible due to the persistence of long-lived, latently infected resting memory CD4+ T cells. The demonstration of a role of the cortical actin in HIV cell-to-cell transfer and infection of memory and naive CD4+ T cells may provide a mechanistic understanding of viral infection and pathogenesis. The higher HIV antigen capture and the broader pattern of migration of memory CD4+ T cells [9] may contribute to a more efficient dissemination of infection that in turn are coupled with additional changes in the cortical actin during the migration to of these HIV-antigen loaded primary CD4+ T cells would also favour infection [48]. Thus, defining the restriction imposed by the cortical actin during HIV infection in different T cell subtypes and how the virus overcomes this constraint specifically in naturally resistant resting CD4+ T cells may be relevant for understanding the pathogenesis of HIV and for the development of new drug therapies.
